# A harmonized dataset of sediment diatoms from hundreds of lakes in the northeastern United States

**DOI:** 10.1038/s41597-022-01661-3

**Published:** 2022-09-03

**Authors:** Marina G. Potapova, Sylvia S. Lee, Sarah A. Spaulding, Nicholas O. Schulte

**Affiliations:** 1 Academy of Natural Sciences, Department of Biodiversity, Earth and Environmental Science, Drexel University, Philadelphia, USA; 2 U.S. Environmental Protection Agency, Office of Research and Development, Center for Public Health and Environmental Assessment, Washington, D.C. USA; 3 Institute of Arctic and Alpine Research, University of Colorado, Boulder, CO USA

**Keywords:** Biodiversity, Limnology, Freshwater ecology

## Abstract

Sediment diatoms are widely used to track environmental histories of lakes and their watersheds, but merging datasets generated by different researchers for further large-scale studies is challenging because of taxonomic discrepancies caused by rapidly evolving diatom nomenclature and taxonomic concepts. We collated five datasets of lake sediment diatoms from the Northeastern USA using a harmonization process which included updating synonyms, tracking the identity of inconsistently identified taxa, and grouping those that could not be resolved taxonomically. Each harmonization step led to an increase in variation explained by environmental variables and a parallel reduction of variation attributable to taxonomic inconsistency. To maximize future use of the data and underlying specimens we provide the original and harmonized counts for 1327 core samples from 607 lakes, name translation schemes, sample metadata, specimen museum locations, and the Northeast Lakes Voucher Flora, which is a set of light microscope images grouped into 1154 morphological operational taxonomic units. Post-hoc harmonization enables data quality control when other approaches (e.g., upfront management of taxonomic consistency) are not possible.

## Abstract (summary)

**Table:** 

Measurement(s)	diatom taxa • abundance
Technology Type(s)	observation • literature review • digital curation
Sample Characteristic - Organism	Bacillariophyta
Sample Characteristic - Environment	Lakes
Sample Characteristic - Location	Northeastern Unites States of America

## Background & Summary

Siliceous remains of diatoms preserved in sediments are commonly used to track environmental change in lakes and their watersheds^1^. In the Northeastern USA, sediment diatoms have been analyzed as part of several regional and national lake surveys carried out by federal and state environmental agencies since 1980s. Diatom count data obtained in the course of these projects were utilized to study lake acidification^2–7^, explore biodiversity patterns^8–10^, develop water-quality and watershed disturbance indicators^11–14^, and estimate the extent of lake cultural eutrophication^15–20^. Numerical models developed from some of these datasets were applied in the past to sediment core data of several lakes to reconstruct their detailed environmental histories^21–25^.

The lake sediment diatom datasets are rich in information that can be further explored for the purposes of environmental inference and for studying diatom ecology and biogeography, but rapid changes in diatom taxonomy and nomenclature over the last decades made them largely incompatible with each other. This led to massive amounts of diatom data produced in the past either not being considered for the inclusion in data analyses or used naively without due attention to taxonomic discord. Our goal was to facilitate future use of the data and underlying specimens for five such datasets by developing a harmonization scheme to ensure compatible data. Most of the diatom samples were surface and bottom intervals of relatively short cores intended for a so-called “top-bottom” analysis of trends. The resulting dataset includes counts for 1327 diatom samples from 607 lakes of the Northeastern United States collected by federal and state agencies from 1991 to 2018 and associated sampling site, sample, and slide information.

Taxonomic discord is a primary source of difference in data because diatom taxonomic concepts are fluid, insufficient, and often contradictory^26^. Lack of stable and universally accepted taxonomic concepts in many groups of organisms is known for its negative impacts on both fundamental and applied research that uses taxonomic data^27,28^ but is especially problematic in microscopic organisms such as diatoms that have relatively few diagnostic morphological characters. Several remedies have been suggested for reducing taxonomic consistency in future diatom projects^26,29^, but post-hoc harmonization is necessary for collating already existing datasets produced by different researchers. It has also been shown to reveal stronger associations between diatom assemblage composition and environmental characteristics^30^. To tackle taxonomic inconsistencies in the selected lake datasets of the Northeastern USA, we used a tiered approach to harmonization. First, we translated originally reported names to their currently accepted nomenclatural synonyms. Second, we revised name usage in subsets of data generated by different laboratories by identifying known taxonomic synonyms. We inspected diatom slides, associated images, and identification sources used in corresponding projects to determine the names applied to the same taxa across different datasets, thus representing de-facto taxonomic synonyms. When examination of identification sources, slides, and images proved impossible to reconcile taxonomic concepts for morphologically similar taxa, they were grouped into “species complexes”. Third, we used Indicator Species Analysis to identify names that were inconsistently applied among data subsets and that caused major disagreements among these subsets and applied additional grouping for those taxa. Using a subset of count data for surface sediment samples and corresponding environmental data, we show that each subsequent harmonization step led to the increase of the amount of variation in species data explained by environmental variables and a parallel decrease of the variation attributable to taxonomic inconsistency as estimated by partial constrained ordination analyses.

Post-hoc harmonization, such as employed here, inevitably leads to a loss of taxonomic resolution^26,30^. As such, documentation associated with each harmonization step should itself be regarded as data. This information on the discrepancies in taxonomic concepts and/or insufficient taxonomic knowledge can be used for future studies aimed at resolving diatom taxonomy and ecology and standardizing morphology-based diatom identification. To enable further investigations of the count data and underlying physical specimens we - therefore - provide the original and harmonized counts, name translation scheme, sampling site information, and specimen museum location data. An illustrated Northeast Lakes Voucher Flora, a compendium of images representing diatom specimens from samples included in this dataset, is provided as another data source and as means of promoting taxonomic consistency of diatom count data generated in the future.

## Methods

### Data sources

Five diatom paleolimnology datasets with sample-specific counts and lake characteristics from 8 states (Connecticut, Maine, Massachusetts, New Jersey, New Hampshire, New York, Rhode Island, Vermont) were downloaded from the online repositories of the United States Environmental Protection Agency (EPA) and from the University of Colorado Institute of Arctic and Alpine Research (INSTAAR) websites, or obtained from the State Agencies, EPA staff, and by querying the internal databases of the Academy of Natural Sciences of Drexel University (ANS) (Table 1). These datasets were chosen because count data could be verified by examining the permanent diatom slides that correspond to counts and have been archived at the Diatom Herbarium of the ANS. Most samples were collected as part of the “top-bottom” sediment sampling scheme that was first adopted in the Paleoecological Investigation of Recent Lake Acidification (PIRLA-II) Project^31^. This type of sampling involves collecting a relatively short (~20–50 cm) sediment core and retaining only the “top” and “bottom” intervals. The exact core depth of the “top” and “bottom” samples varied among projects and cores: “top” intervals were either 0–1 cm, 0–2 cm, 0–0.5 cm or 0.5–1 cm, but mostly the same within each dataset, while the “bottom” intervals were not standardized and usually the lowermost for each core. The “top” samples are thought to represent present-day lake conditions, as they typically contain diatoms accumulated within the few years prior to sampling, whereas the bottom intervals from >30 cm deep in the cores from natural lakes are considered as representing pre-industrial (i.e., pre-1850) conditions^12^. All datasets included both natural and artificial lakes.

**Table 1:** Data sources.

Dataset code	Geographic coverage	Diatom Counts	Sampling site characteristics	Sampling period	Number of samples
EMAP	Northeastern USA	^35^	^35^	1991–1996	603
NLA07	Northeastern USA	^58^	^58^	2007	238
NLA17	Northeastern USA	^53^	^54,59^, Vermont Department of Environmental Conservation	2017	106
VT	Vermont state, USA	Vermont Department of Environmental Conservation	^54^, Vermont Department of Environmental Conservation	2012–2018	178
NJ	New Jersey state, USA	Academy of Natural Sciences of Drexel University	New Jersey Department of Environmental Protection	2005–2014	202

The EMAP dataset was generated as part of the Environmental Monitoring and Assessment Program-Surface Waters (EMAP-SW) lake survey aimed at estimating extent and geographical distribution of lakes and their ecological condition^32,33^. The design of EMAP-SW was based on a systematic grid of randomly spaced points, so that conditions and trends could be estimated with known uncertainty^34^. Lakes were sampled by the EPA, and some lakes were sampled twice per year and/or across multiple years. The details of coring, water sampling, and diatom sample processing methods are described by Dixit *et al*.^12^. Water-quality, lake, and landscape data associated with EMAP samples are publicly available^35^, while lake depth data were retrieved from an internal EPA archive. Sampled lakes were at least 1 m deep and had surface area of at least 0.01 km^2^.

The NLA07 dataset is a subset of data collected as a part of the National Lakes Assessment survey (NLA) of 2007, which was conducted by the EPA^36^. The survey used a probabilistic sampling procedure to select lakes representative of the population of lakes in the conterminous United States larger than 0.04 km^2^ in area, at least 1 m deep, and having at least 0.001 km^2^ of open water. Lakes were sampled once in 2007.

The NLA17 diatom samples were collected by state agencies following EPA field protocols^37^ and mostly from lakes surveyed by EPA in 2017. Sampled lakes had surface area of at least 0.01 km^2^. All diatom samples represent surface sediments (i.e., “top” samples only) and were processed and enumerated as part of a cooperative agreement between the EPA and INSTAAR. The enumeration procedure for NLA17 was different from all other datasets, as three analysts used a pre-count voucher flora based on morphological operational taxonomic units (OTUs) as recommended by Tyree *et al*.^26^ for improving taxonomic consistency. For reporting purposes, these OTUs were translated to currently accepted taxa names. Environmental data were collected by EPA within the NLA framework in 2017, but for several lakes from Vermont and New York the water-quality data were collected by corresponding state agencies.

The Vermont (VT) dataset includes samples collected by the Vermont Department of Environmental Conservation (VDEC) following EPA methodology^37^. Some lakes were sampled once, but some others in multiple years. The analyst who originally enumerated the diatoms in VT samples revised the diatom count data to match the taxonomy and OTU codes used in NLA17 voucher flora.

The New Jersey (NJ) dataset consists of samples collected by the New Jersey Department of Environmental Protection (NJDEP). The ANS processed and enumerated the NJ samples following protocols by Charles *et al*.^38^. This dataset includes samples from the cores intended for the “top-bottom” analysis and two cores with multiple intervals (Greenwood and Surprise Lakes), but there were some lakes with data only from surface sediment samples.

Original count data consist of sample codes, taxa names as reported in the source file of each dataset, and the total number of counted valves per sample. For most samples, approximately 500 diatom valves were counted, but some samples were sparse and therefore fewer specimens were recorded by the original analysts. When possible, we used the same sample identifiers as in the original source. Several sample codes as reported in the EMAP source file were found to be non-unique, as both “top” and “bottom” samples were listed under the same sample identifier. To create a unique sample identifier for EMAP samples, we concatenated contents of the following fields: “LAKE_ID”, “YEAR” (year of sampling), “VISIT_NO” (visit number) and “COREPOS” (“top” or “bottom” position of the sample in the core). In the VT dataset no unique sample identifiers were provided, therefore they were created by concatenating lake names, year of sampling, and “top” or “bottom” designation.

### Taxonomic harmonization

**Fig. 1:**
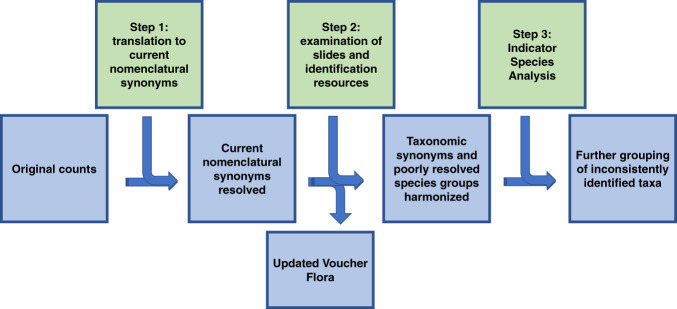
Taxonomic harmonization workflow.

Originally reported names were first translated to their current nomenclatural synonyms (Fig. 1, Step 1). As there is no universally accepted list of current diatom names, determination of which synonym is “current” was based on our knowledge of freshwater diatom taxonomy and with consultation of several contemporary diatom floras^39–46^ and online resources such as Diatoms of North America^29^, Algaebase^47^, and DiatomBase^48^. For names listed in DiatomBase, we report their unique identifiers known as Aphia IDs to link taxa names to names’ authors, publication details, and other nomenclatural information. Each dataset included considerable number of provisional names as some species were not yet formally described at the time of sample analysis or could not be identified to species level for other reasons. These names were left unchanged at this step.

At the next harmonization level (Step 2), we revised name usage in subsets of data by identifying known taxonomic (subjective) synonyms, i.e., names based on different nomenclatural types, but applied to identical species. Information sources used for reconciling taxonomic synonyms are cited in (Harmonization_102421.xlsx). We also inspected diatom slides, as well as associated images and identification sources to determine which names were applied to the same taxa in different datasets.

When examination of identification sources, slides, and images failed to reconcile taxonomic concepts used by various analysts for morphologically similar taxa, they were grouped into “species complexes” or “slash groups”^30^.

For the final harmonization level (Step 3), Indicator Species Analysis was applied to the harmonized count data from Step 2 to identify names that caused major remaining disagreements among datasets^49^. Taxa names with the most significant indicator values (p = 0.001) were considered as candidates for additional grouping. Samples from the datasets where these taxa were most abundant were examined to determine whether inconsistencies were caused by misidentifications or by objective difficulties in separating these taxa given the information available in standard identification resources. In the latter case, the taxa were further grouped into “slash groups”, which sometimes corresponded to genus-level identification (e.g., all taxa within the genera *Discostella, Tabellaria* and *Rossithidium*) or combined several species of the same or different genera (e.g., all species of *Staurosira* and *Staurosirella* except those with salient morphological features and therefore easy to separate).

The reduction of taxonomic inconsistency by harmonization was quantified by Redundancy Analysis (RDA) and visualized using non-metric multidimensional scaling (NMDS)^50^. Both RDA and NMDS used Hellinger-transformed diatom count data^51^ and were carried out with R *vegan* package^52^ with R code archived together with the dataset (DiatomHarmonization_032222.R).

### Voucher flora

To facilitate future use of the count data and to promote taxonomic consistency, we provide images of the most common diatoms found in Northeastern Lakes compiled in a Voucher Flora. This set of light microscope images is an updated version of the Voucher Flora produced in 2018^53^. All images were captured by digital cameras installed on light microscopes equipped with differential interference contrast optics and 100x oil 1.4 numerical aperture objectives. Several new OTUs were added as we examined slides during this study, and a few additional images were added to the existing OTUs to better illustrate corresponding diatoms taxa. Additionally, the concepts of several OTUs were refined by splitting or merging them. The translation scheme shows scientific names of taxa that correspond to OTUs when they could be determined or provisional names of the taxa when morphological characters of OTUs were sufficiently different from any established taxa.

### Sample and slide data

To assemble sampling site information, we used published and unpublished data from sources listed in Table 1. Sampling site information includes lake name, geographical coordinates, state name, lake surface area, and depth. The lake depth data in most cases represent depths at the sampling sites, which were designed to be located as close as possible to the deepest points of the lakes and thus can also be considered an approximation of maximum lake depths. In the NLA07 dataset, the variable DEPTHMAX (maximum lake depth) was used as it had practically the same values as “DEPTH_X” (sampling site depth) but had data for a higher number of lakes than the latter. For a few samples in the NLA17 dataset that lacked lake morphometry data, surface area and depth were retrieved from Conservation Gateway^54^ and the LAGOS dataset^55^. Unique identifiers of lakes (COMID) were available for some datasets, while for others COMIDs were retrieved from the National Hydrography Dataset^56^. As some lakes had multiple COMIDs and others had none, we created unique lake identifiers by concatenating COMIDs (if available, and choosing the COMID closest to the sampling site coordinates if multiple were available) and lake names.

Sample-specific information includes collection date, as well as the depth of the core and the thickness of the sampled core interval. Permanent diatom slides made from the samples that constitute this dataset are housed at the ANS Diatom Herbarium (collection code ANSP). At least one, but usually two and occasionally more replicate slides for each of the 1224 (92.2%) samples are accessioned in the collection.

## Data Records

The Data Records are stored in the U.S. EPA ScienceHub data repository^57^ and are publicly available at 10.23719/1524246. The Dataset consists of the following components:

1. **Counts_original_long_102421.xlsx:** this file contains original diatom count data in long format.
2. **Harmonization_102421.xlsx** is the taxonomic harmonization scheme with notes and references.
3. **SiteInfo_031922.xlsx** contains sampling site- and sample-level information.
4. **DiatomHarmonization_032222.R** is an R code that uses the abovementioned files as input to harmonize original diatom counts.
5. The OUTPUT folder contains five Comma Separated Values (.csv) files representing original (**Counts_1327_wide.csv**) and harmonized diatom count data in wide format. (**Step1_1327_wide.csv**, **Step2_1327_wide.csv**, **Step3_1327_wide.csv**), and the summary of the Indicator Species Analysis (**INDVAL_RESULT.csv**).
6. **Slide_accession_numbers_102421.xlsx** has slide accession numbers at the ANS Diatom Herbarium.
7. The Voucher Flora is documented by diatom images compiled into plates (**NE_Lakes_Voucher_Flora_102421.pdf)** and the equivalency between Operational Taxonomic Units (OTU) codes that accompany diatom images in the Voucher Flora and diatom scientific or provisional names with identification sources, references, and notes (**VoucherFloraTranslation_102421.xlsx**).

## Technical Validation

**Fig. 2:**
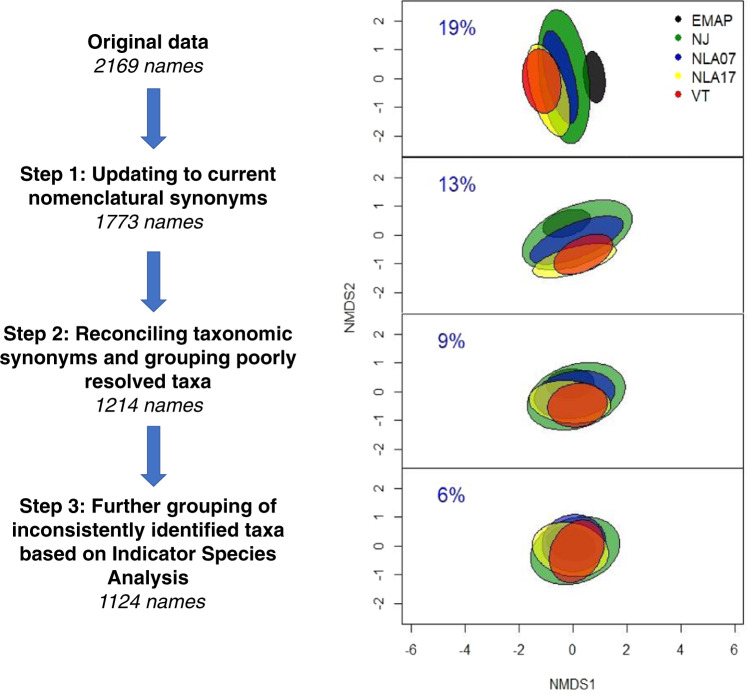
Assessment of the degree of taxonomic inconsistency among the 5 subsets of diatom count data by Redundancy Analysis. The percent of total variation in the count data explained by “dataset” is shown in each panel and visualized by confidence ellipses in the NMDS ordination space.

The RDAs of the entire dataset of 1327 diatom counts at four stages of harmonization demonstrate a decrease of variation in diatom data explained by dataset identity from 19% in the original count data, to 13% at harmonization Step 1, 9% at Step 2, and 6% at Step 3. The overlap of confidence ellipses in the non-metric multidimensional scaling (NMDS) plots illustrates increased similarity among data subsets at each successive harmonization level (Fig. 2).

**Fig. 3:**
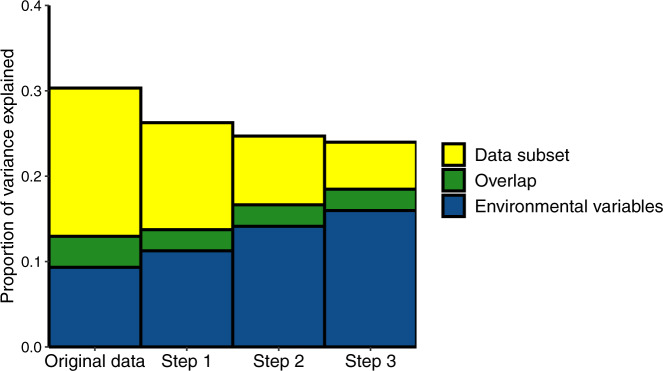
Proportion of variation in diatom count data explained by measured environmental characteristics (blue) and taxonomic differences among five count subsets (yellow) as measured by partial RDA analyses. Green color shows the overlap in variation explained simultaneously by both environmental and taxonomic differences: this part of the variation is attributable to environmental differences among datasets.

As differences in diatom assemblages among datasets may be driven by both taxonomic discrepancies and environment, a series of partial RDAs was carried out with a subset using all 560 counts representing surface sediment samples from 472 lakes for which water-quality data were available. Environmental parameters (Supplementary File 1: WaterQualityData_021822.xlsx) used in these RDAs were site latitude and longitude, lake surface area and depth, and six water-quality characteristics which were previously found to be important drivers of diatom distribution in Northeastern lakes:^11^ pH, conductivity, and four variables related to lake nutrient status: concentrations of chlorophyll *a*, total phosphorus, total nitrogen, and Secchi disk depth. Water-quality data were retrieved from sources listed in Table 1. For samples with multiple water-quality measurements in the sampling year, values were averaged for each variable, as water chemical composition is known to considerably fluctuate in lakes across seasons. Lake surface area and depth, conductivity, total nitrogen, total phosphorus, chlorophyll *a*, and Secchi disk depth data were log-transformed. For each diatom count dataset, original and three harmonized, two partial RDAs were constructed. One partial RDA was carried out with the dataset as the sole constraining variable and the ten environmental variables as conditional variables. The second partial RDA used the ten environmental variables as constraints and the dataset as a conditional variable. Figure 3 illustrates both the reduction of taxonomic inconsistency and the increase of the variation explained by environmental factors at each subsequent harmonization step.

## Usage Notes

All datasets presented here can be used for addressing a wide range of research questions. The harmonized data are most suitable for elucidating major trends in diatom assemblages and analyses not requiring fine taxonomic resolution. Note that the harmonization process described here does not simply raise taxa IDs to higher taxonomic levels, as this is not an ideal way of addressing taxonomic uncertainty in diatom data. First, nomenclature of diatoms is rapidly changing with new genera being split from the old ones at an unprecedented rate. Second, some taxa, such as small fragilarioid diatoms, may be almost impossible to identify with certainty even at genus level in routine enumeration with light microscopy. At the same time, other taxa within these same genera have unique morphological characters that make their identification at species level straightforward. Therefore, mechanical lumping of species to genera or higher-level taxa sacrifices potential environmental indicators. Data users interested in investigating distribution patterns of individual taxa within problem groups should consult our harmonization notes to understand data caveats and possibly re-inspect physical specimens. The museum slide location data (Slide_accession_numbers_102421.xlsx) are provided here to facilitate such studies.

Users should be aware that some taxonomic disagreement among individual data subsets is present even in our harmonized dataset. This is due to several factors. First, it is impossible to correct occasional misidentifications without complete recounting of each slide, and some degree of taxonomic uncertainty is expected in diatom data for reasons outlined by Tyree *et al*.^26^. We opted not to group some taxa in the last harmonization step that were identified in Indicator Species Analysis as causing taxonomic discord if they have been previously established as reliable indicators of environmental conditions (e.g., common planktonic species in the genus *Aulacoseira*). Second, we were unable to clarify the identity of many originally reported taxa if they were rare and not documented by photographs.

The harmonization scheme (Harmonization_102421.xlsx) can be further modified based on additional taxonomic investigations, while the associated R code (DiatomHarmonization_032222.R) provides a straightforward mechanism for diatom data versioning. The harmonization approach used here can also be adapted for collating other diatom datasets prior to any data analyses that require taxonomic consistency.

The Voucher Flora shows the taxa that have been thus far encountered while enumerating NLA17 samples and reexamining selected slides from the other datasets for harmonization purposes. It includes all taxa that are common in Northeastern lakes and many rare ones, but further sampling campaigns and detailed taxonomic studies are expected to reveal more diatom taxa in this region. The users should therefore consider this flora a work in progress and be aware of the possibility of encountering more species, especially if sampling lakes not represented in the dataset. Although this Voucher Flora has been developed for the lakes of Northeastern United States, it can be used as a taxonomic reference for other regions of North America as many diatom taxa have wide distribution not limited to the region.

The environmental variables used here to test the effectiveness of harmonization are commonly reported as important drivers of sediment diatom distribution in lakes, but they do not exhaust all options for characterizing lakes and their watersheds. Users should be able to obtain additional lake characteristics and other pertinent data from sources and datasets relevant to specific studies, geographic areas, and research questions. There are innumerable ways of summarizing environmental lake data, and therefore the water-quality data and analyses used here to examine the effects of taxonomic harmonization are not recommended as a universal methodology for lake or diatom studies and are presented as supplementary material.

The subsets of data merged here were collected with somewhat different field methods, which may have implications for some studies. For example, the thickness of sampled core interval varies from 0.5 cm to 2 cm with two “bottom” samples being even thicker (SiteInfo_031922.xlsx). Additional metadata and details of sampling programs may have to be retrieved as necessary.

### Disclaimer

The views expressed in this article are those of the authors and do not necessarily represent the views or the policies of the U.S. Environmental Protection Agency. Any mention of trade names, manufacturers or products does not imply an endorsement by the United States Government or the U.S. Environmental Protection Agency.

## Supplementary information

Supplementary File 1 - Water quality data

## Data Availability

R code used to generate harmonized sets of diatom count data and test the described dataset is archived together with Data Records at 10.23719/1524246^57^.
